# Supplementary material for: Quality of life and retention in care among people living with HIV initiated on ART in the era of “Universal Test and Treat” policy at a large HIV Clinic in South Western Uganda
Source: PLoS One. 2025 May 15;20(5):e0324024. doi: 10.1371/journal.pone.0324024 (PMC12080831; doi:10.1371/journal.pone.0324024)
Supplement: Attachment — Submitted filename: Response to Reviewers.docx [file pone.0324024.s003.docx]

# Point-by-point responses to reviewer’s comments

We sincerely thank the Associated Editor, Prof. Kwasi Torpey, for overseeing the review process and the reviewers for their insightful and constructive comments, which have significantly improved the quality of our manuscript. We have carefully addressed all reviewers’ comments and provided detailed responses, indicating exactly how each point has been incorporated into the revised manuscript. We look forward to your favorable response.

# Reviewer #1:

**Strengths**

● Setting of the study is relevant as it is in an area with high HIV burden in Africa so the results are generalizable

● They used a validated tool to measure QoL

● Good description of statistical analysis

● Sample size calculations well described

**Response: Thank you for the encouraging comments.**

Weaknesses

● Wording in the abstract and part of the manuscript is contradictory- looking at the results on line 36 contradicts the findings on line 41

**Response: We agree with this observation. The results and conclusions have been harmonized. The conclusion now reads: “In this cohort of PLWH on ART, those who initiated the treatment at a higher baseline CD4 were less likely to have good mental health-related QoL functioning compared to those initiating at lower CD4 counts. However, the two groups were comparable in physical health-related QoL and retention in care.”**

● The stratification of CD4 count into only <500 and ≥500 cells/μl oversimplifies the immunological response spectrum. It would have been more robust if there were finer CD4 categorization.

**Response: A more granular categorization of CD4 counts could provide deeper insights into associations. However, such disaggregation may lead to information loss and reduced statistical power. Clinically, CD4 count is commonly categorized as <500 and ≥500 cells/µL to reflect immunological status, as lower counts (typically below 500 cells/µL) are associated with WHO clinical stage 3 opportunistic infections. We have therefore maintained the categorization for these reasons.**

● With universal test and start the usefulness of the study is in question

**Response: Universal test and treat (UTT) enables ART initiation immediately after HIV diagnosis, regardless of CD4 count. We thank the reviewer for raising this point, and indeed we are aware it is now the standard of care, and this is the basis of our research question. There are concerns about impact of universal test and test on quality of life (QoL) and retention, as long-term ART exposure may increase the risk of adverse effects, drug toxicities, and interactions, especially among persons initiating ART at higher CD4 counts, i.e. without symptoms of HIV disease. To our knowledge, previous studies have primarily examined QoL among individuals with low CD4 counts. Providing evidence on QoL in those with high CD4 counts has important clinical and public health implications, as discussed in the introduction (page 3, paragraphs 2–3).**

● Only 81 (27%) of the participants responded to the mental health QoL domain with 277 responding to the physical health QoL however the authors did not explain the reason for the variance calling into question selection bias and the validity of the results

**Response: We are grateful for this comment and this non-response has been reported in the results section previously. In the revised manuscript, we have acknowledged the non-response rate as one of the study limitations. The new text reads as follows:**

**“Not all participants responded to the physical and mental health-related quality of life (QoL) domain questions, including a few independent variables resulting in missing data. This non-response may introduce selection bias, reduce statistical power, and limit the generalizability of the findings. Given its potential to compromise the reliability of the results, non-response must be carefully considered when interpreting the data.**

● The definition of retention (active in care ≤7 days after appointment vs. lost to follow-up >7 days after appointment) is quite strict compared to what we would have expected them to use e.g., WHO, definitions (often 30-90 days). The authors should justify this definition and discuss how it might affect comparability with other studies.

**Response: We agree that a strict definition was used in the present study compared to previous studies and this may have led to overestimation. The timeline is appropriate in our study as the maximum number of days elapsed following the scheduled appointment was just 20 days. This suggests that the 30-day rule was not applicable in the study. We have acknowledged this in the limitations section. The text reads as follows:**

**“Moreover, we used a shorter time frame to measure retention compared to previous studies, which may have led to an overestimation of retention rates.** **However, the shorter time frame is more relevant to our study, as the maximum delay past a scheduled appointment was only 20 days”.**

● The authors conclude that PLWH with higher baseline CD4 counts have lower mental health-related QoL, but the adjusted odds ratio of 0.56 (95% CI 0.32-0.97) has a confidence interval that nearly includes 1, suggesting a relatively weak association. This should be acknowledged in the discussion. This could be attributed to the sample size

**Response: We agree. A new text regarding the attenuated association has been added and it reads:**

**“Although the association is borderline statistically significant, the finding that PLWH with a higher baseline CD4 count are less likely to report good mental health-related QoL compared to those with a lower baseline CD4 count may be attributed to ART-related side effects.”**

● In lines 268-269, the authors report that the study findings concur with previous reports of , “ significant decrease in mental disorders among PLWH and but mild to moderate cognitive impairment remains high at all stages of HIV infection “ the results presented do not support this conclusion as the MH QoL does not specify what was assessed and the outcomes of the assessments.

**Response: This sentence has been deleted**.

● Authors should use more graphs and tables to describe/discuss the results

**Response: Thank you for your suggestion to use more graphs and tables to describe and discuss the results. We have presented our results in four tables and believe this represents the average number of tables in a manuscript. However, we are open to revisiting this if specific results could be more effectively conveyed through graphs.**

● While the study adjusted for several variables, other critical confounders such as ART adherence, duration on ART, ART regimen type, and socioeconomic factors were not included in the model.

**Response: We agree that there are unmeasured confounders and this has been included in the study limitations section. The text reads as follows:**

**“Potential unmeasured confounders such as ART adherence, duration, regimen type, and socioeconomic factors, were not included in the study and should be considered when interpreting the results.”**

● Language

○ Some sentences are excessively long, making comprehension difficult.

**Response: We have re-checked and shortened longer sentences.**

○ Terms such as "CD4 count at ART initiation" and "baseline CD4 count" are used interchangeably. They should standardize the language

**Response: Thank you for the comment. In the revised manuscript, we used “Baseline CD4 count”.**

○ The discussion section reiterates findings without substantial analysis

**Response: We have revised most texts in the discussion to ensure substantial analysis.**

○ There are occasional grammatical errors and awkward phrasings that could be improved.. Generally more proofreading is needed

**Response: We have proofread the manuscript and corrected all grammatical errors and awkward phrasings.**

**Minor concerns**

● In Table 1, there are some discrepancies in the total numbers for variables like WHO clinical stage (n=275) and most recent CD4 count (n=272) compared to the total sample (n=300). These missing data should be explained.

**Response: We acknowledge that some data is missing, and this issue has been addressed in the limitations section. For more details, please refer to our response to comment #4.**

● Looking at the table some numbers do not add up, WHO clinical stage 3 for example

**Response: This has been addressed in the responses above.**

● The authors report mean physical and mental health-related QoL scores in Table 2, but it's unclear how these scores should be interpreted (e.g., what is the possible range, what constitutes a clinically significant difference).

**Response: The physical and mental health-related QoL scores based on the Medical Outcomes Study-HIV Health Survey (MOS-HIV) tool range from 0 to 100. We added the statement 'The scores range from 0 to 100' under the 'Variables and Measurement' sub-section in the Methods and Materials section. Since the scores are numerical, we assessed whether statistically significant differences in mean scores existed based on baseline CD4 count categorization. To our knowledge, no established threshold exists for defining a clinically significant difference in mean scores.**

**Suggestions**

1. Provide a thorough explanation for the low response rate on the mental health-related QoL domain and discuss the implications for the validity of the findings.

**Response: We have addressed the non-response rate and included its implication on the overall results.**

2. Analyze and report the association between time since ART initiation and QoL outcomes.

**Response: We are grateful for this comment. The time since ART initiation was included as a covariate in the analysis of the relationship between baseline CD4 count and QoL. Please see the footnote in Table 3.**

3. Include a more detailed description of the MOS-HIV tool, including the range of possible scores and what constitutes a clinically meaningful difference.

**Response: There is no universally agreed-upon threshold for a clinically meaningful difference in scores from the Medical Outcomes Study-HIV Health Survey (MOS-HIV). However, based on similar health-related quality of life (HRQoL) instruments, researchers often consider a difference of 5–10 points to be clinically significant. In response to the comment, we have added the text below in the methods section:**

**"While no universally accepted threshold exists for a clinically meaningful difference in MOS-HIV scores, prior research on similar Health-Related Quality of Life (HRQoL) tools suggests that differences in the range of 5–10 points may be considered clinically significant."**

4. Include data /scores of the different domains assessed using the QoL tool and assess explore associations between CD4 accounts and the different domains

**Response: We chose to present the summary scores as they are simpler, rather than the individual specific domains as they are over 10 and we did not have specific hypothesis on differences among the domains.**

5. Address potential selection bias, as those retained in care until the interview period might have different characteristics than those lost to follow-up.

**Response: We recognize some PLWH are lost to follow-up during care, before they were interviewed. However, given that retention was similar by level of CD4 count at treatment initiation, suggests that selection bias may not play a significant role.**

6. Consider conducting sensitivity analyses with different definitions of retention to assess the robustness of the findings.

**Response: The maximum delay past a scheduled appointment was only 20 days in our study so we did not perform a sensitivity analysis based on a much shorter day.**

7. Discuss the implications of the findings more thoroughly, particularly how mental health interventions might be integrated into HIV care programs for those initiating ART at higher CD4 counts.

**Response: We have provided additional text around the implications of findings and it reads:**

**“Given the association between higher baseline CD4 counts and poorer mental health-related QoL, HIV care programs should incorporate mental health services at the point of ART initiation. The screening results would then guide referrals to mental health professionals for further assessment and intervention. Cognitive Behavioral Therapy (CBT)-based interventions may be incorporated into pre-ART counseling sessions to help PLWH cope with the psychological impact of HIV diagnosis and treatment initiation, especially for those initiating ART at high CD4 count. Additionally, peer support groups may help normalize the experience of initiating ART at higher CD4 counts, as this has been shown to reduce stigma and feelings of isolation. Health education on ART adherence and mental health should be included in HIV treatment literacy programs. Implementation research may be needed to evaluate the effectiveness of different mental health integration models in improving both mental health outcomes and retention in care. Adopting a comprehensive and patient-centered approach by the healthcare system may improve both mental health outcomes and long-term engagement in HIV care.”**

# Reviewer #2:

1. The manuscript is generally well-organized, with a clear structure and logical progression. The introduction provides a solid background, the methods are detailed, and the discussion contextualizes the findings. However, there are some concerns about the robustness of the data analysis and the conclusions drawn:

a. The manuscript introduction provides a good background but lacks a clear research gap statement.

**Response: Thank you for this comment. We have re-checked the introduction section and have re-affirmed the problem statement/research gap as indicated in the introduction section (paragraph 4, page 4). The gap reads:**

**“Therefore, it is not completely clear whether ART initiation at a higher baseline CD4 count might lead to negative effects on QoL or retention compared to ART initiation at a lower baseline CD4 count in PLWH in resource-limited settings such as Uganda”.**

b. The historical progression of HIV treatment guidelines is informative but too detailed given HIV has been with us for decades and some of it could be summarized in a table.

**Response: We have summarized the information about the HIV treatment guidelines. We aim to provide clear information about changes in treatment guidelines and how this has impacted care delivery, which is important in understanding the need for the current research.**

c. The topic is relevant, though similar studies have been conducted in other settings. The manuscript would benefit from emphasizing how this study adds new insights beyond existing literature.

**Response: Previous studies have examined QoL at lower CD4 counts. Therefore, the relationship between ART initiation at a higher baseline CD4 count and QoL or retention in resource-limited settings such as Uganda remains completely unclear. Our study filled this gap and this evidence has important clinical and public health implications as discussed in the introduction (page 3, paragraphs 2–3).**

d. The justification for using the specific QoL measurement tool (MOS-HIV) should be strengthened given there are a number of tools, so important to justify why this specific tool was utilized.

**Response: We have included a text summarizing the rationale for using the MOS-HIV tool for QoL measurement on page 5 (variable and measurement sub-section).**

e. The methods section is concise but could explicitly mention sample size justification. It is not clear how the 300 sample size was arrived at. Were all clients enrolled in the two years enrolled or only those with baseline CD4?

**Response: The sample size determination was hypothesis-driven, as described on page 6 in the sample size calculation section. Given that this was an analytical study, we powered the study at 80% to detect the anticipated mean difference between the comparison groups. The two-year period provided the sampling frame; however, not all individuals within this timeframe were included, as we used consecutive sampling. For each sampled individual, we retrieved baseline CD4 count data. These issues have been explained in the respective sections of the methods.**

f. From the results and discussion, the primary finding, that higher CD4 count at ART initiation is associated with lower mental health-related QoL, is not fully explored. The authors speculate that this is due to ART-related side effects, but no direct evidence such as qualitative interviews or neuropsychiatric assessments is provided to support the claim.

**Response: We do not have qualitative data to complement the findings. This may be a study limitation as already addressed in the study strengths and limitations section on page 15. In the revised manuscript, we provided biologically and socially plausible explanations for the findings on page 12, paragraph 1. The text reads:**

**“A higher baseline CD4 count is often associated with a less advanced stage of HIV and better physical health. However, individuals with higher CD4 counts may still face significant psychological challenges. Despite their relatively better physical health, they may struggle with the emotional and psychological implications of living with HIV and lifelong treatment. These individuals may experience heightened anxiety about disease progression, uncertainty regarding their long-term health, or the psychological burden of long-term ART. Moreover, they may be less likely to perceive themselves as “sick,” potentially diminishing their sense of urgency about adhering to ART. This could create a paradox, where they feel less concerned about their health overall, even though HIV still requires ongoing management. Conversely, those with lower CD4 counts may have already encountered more severe health issues, which could foster better-developed coping strategies. Such individuals may have a clearer understanding of their health status and the critical need for adherence to ART, which could contribute to their enhanced mental well-being. Being more attuned to the immediate necessity of medication and healthcare, they may adopt a more proactive approach to health management, including seeking psychosocial support, thereby improving their overall mental health. Our findings thus highlight the significance of using appropriate screening tools and effective treatments for mental health issues among PLWH, the prioritization of the diagnosis and treatment of mental health disorders, and the integration of mental health screening and QoL measures in HIV programming.”**

g. Retention in care is analyzed, but the sample size is relatively small (n=300), compared to the clients in the clinic and retention is assessed over a relatively short period. A longer follow up period would give more reliable conclusions. Further, would be good to look at different time periods like 3, 6, 12 ,24 months retention.

**Response: The study’s sample size was calculated based on sound scientific methods so is sufficient to detect the desired associations in the sample. We examined retention over 20 months and now provided the median retention time by baseline CD4 categorization to improve the interpretation. The new text reads:**

**“The median retention time was 17 months (IQR 8-19) for individuals with baseline CD4 < 500 cells/cm³ and 18 months (IQR 9-20) for those with baseline CD4 ≥ 500 cells/cm³.” These data have been included in the results in Table 2.**

**Also, since 20 months of follow-up may not be long enough, we added the relatively shorter period as one of the study’s limitations. The text reads: “We studied retention over 20 months of follow-up and this may have not provided accurate results on its relationship with baseline CD4 count. A relatively longer follow-up time may be required in future studies”**

h. There is no significant difference in physical health-related QoL or retention, but the authors still discuss the need for interventions to improve retention. While this is relevant, the justification based on the data provided is weak.

**Response: We agree that retention was not associated with baseline CD4 count in the multivariable analysis. As already discussed, a higher proportion of PLWH who initiated ART at a lower baseline CD4 (<500 cells/ul) were retained in care compared to those who initiated ART at a higher baseline CD4 (≥500 cells/ul) in the bivariate analysis. We discussed the consistency of our findings with previous study results and provided a few discussions around approaches to improve/maintain retention in care because it is the cornerstone for successful ART delivery.**

i. The categorization of CD4 count as <500 vs. ≥500 cells/µl used in the study is somewhat arbitrary. The manuscript references cut offs of 200, and 350 yet settles on 500 without a specific explanation why the 500 cut off. Especially given that quality of life at the different CD4 cut offs has been shown to be different including risk of those below 200 having high risk of advanced HIV disease compared to those above, sensitivity analyses using different cutoffs (e.g., <200 vs. ≥200, <350 vs. ≥350 ) could strengthen the findings.

**Response: We have performed a sensitivity analysis and the results are presented in Table 4. We categorized baseline CD4 count into <200 vs. ≥200 cells/µL and <350 vs. ≥350 cells/µL to assess differences in retention and quality of life (QoL).**

**No significant differences were observed in these outcomes at these thresholds, suggesting that any differences in outcomes may only be apparent at higher CD4 count thresholds.**

j. The results section is repetitive—avoid re-explaining numbers already presented in tables.

**Response: We have revised the results section by removing certain absolute figures and providing the overall number (denominator) where applicable. Since this is a quantitative study, we aim to present the findings in numerical terms rather than using qualitative descriptors such as 'the majority,' 'most participants,' or 'few participants’.**

k. The authors should discuss alternative explanations for their findings, particularly regarding mental health-related QoL.

**Response: We have provided alternative explanations regarding the mental health-related QoL. Please refer to our response to comment number “f” above. Please, also see page 12, paragraph #1.**

l. There seems to be aspects of overinterpretation of results e.g the mental health QoL findings are interesting but should be cautious in attribution. The conclusion that higher CD4 count at ART initiation leads to lower mental health-related QoL is not fully supported by the data. Other attributes such as socioeconomic factors, mental health comorbidities, or ART side effects should be considered.

**Response: We have revised the conclusion to avoid overinterpretation and it reads:**

**“Our study showed that ART initiation at baseline CD4 count ≥500 cells/µl compared to baseline CD4 count <500 cells/µl is associated with a lower likelihood of good mental health-related QoL but similar good physical health-related QoL and retention in care.”**

m. The conclusions in the manuscript align with the results, be more direct and linked to the study’s aims and findings.

**Response: We have made the conclusion more direct as stated here:**

**“In this cohort of PLWH on ART, those who initiated the treatment at a higher baseline CD4 were less likely to have good mental health-related QoL functioning compared to those initiating at lower CD4 counts. However, the two groups were comparable in physical health-related QoL and retention in care.”**

n. There’s need to have the policy implications brought out clearly and recommendations for healthcare interventions based on findings. The authors discuss mental health interventions for early ART initiators, but specific recommendations are missing.

**Response: This concern was equally raised by reviewer #1. We have described specific mental health interventions in the revised manuscript on pages 15 to 16 and it reads as follows:**

**“Given the association between higher baseline CD4 counts and poor mental health-related QoL, HIV care programs may incorporate mental health services at ART initiation. The screening results would then guide referrals to mental health professionals for further assessment and intervention. Cognitive Behavioral Therapy (CBT)-based interventions may be incorporated into pre-ART counseling sessions to help PLWH cope with the psychological impact of HIV diagnosis and treatment initiation. Additionally, peer support groups may help normalize the experience of initiating ART at higher CD4 counts, reducing stigma and feelings of isolation. Health education on ART adherence and mental health should be included in HIV treatment literacy programs. Implementation research may be needed to evaluate the effectiveness of different mental health integration models in improving both mental health outcomes and retention in care. Adopting a comprehensive and patient-centered approach by the healthcare system may improve both mental health outcomes and long-term engagement in HIV care”**

o. No mention of study limitations yet important to understand if any potential biases.

**Response: We described the study's strengths and limitations in our earlier submission but have expanded them in the revised manuscript. We have a dedicated sub-section “Study strengths and limitations” to ensure easy readability.**

2. The authors have done a good analysis and used appropriate statistical models (logistic regression for QoL outcomes and Cox proportional hazards for retention). However, there are some concerns:

a. The assumptions for the Cox model (proportional hazards) are not explicitly tested. The authors should test proportional hazards assumptions for the Cox model.

**Response: We tested the assumptions of the Cox model (proportional hazards). In the methods section on page 7, we stated "We used Schoenfeld residual testing to check the assumptions of the Cox Proportional Hazard regression model for each variable. The null hypothesis was the model is correctly specified so p>0.05 was taken to mean no violation of the assumptions."**

**In the result section (page 10), we now confirm that the assumption was not violated. The new text reads as follows: "Schoenfeld residual testing confirmed that the assumptions of the Cox Proportional Hazards regression model were upheld, as all p-values were greater than 0.05."**

b. The logistic regression model does not report any goodness-of-fit tests. This is necessary to confirm that the model is valid.

**Response: On page 10, we have now reported the goodness-of-fit test statistics for the logistic regression, confirming the model was valid. In the methods section (Page 7), we have stated in the methods section that model fit was tested and the text reads:**

**“To evaluate the goodness-of-fit of the logistic regression model, we applied the Hosmer-Lemeshow test. The null hypothesis was that the model provides an adequate fit to the data, while the alternative hypothesis was that the model does not adequately fit the data.”**

**We have provided findings of the model fit in the results section and it reads:**

**“The Hosmer-Lemeshow test for goodness-of-fit demonstrated that the model adequately fit the data for the physical QoL (χ² = 29.14, p=0.085) and the mental QoL (χ² = 113.02, p= 0.8557), indicating no significant discrepancy between the observed and predicted outcomes.”**

c. The statistical analyses are generally appropriate, but consider analyzing retention further with additional sensitivity checks e.g., competing risks analysis for mortality among those with very low CD4 counts vs. loss to follow-up especially those with high CD4s with the perception of “being well”.

**Response: We agree with the reviewer about the use of competing risks analysis. Given that retention was a secondary outcome, we believe this analysis is beyond the scope of this paper and will be explored in a separate paper as a primary outcome.**

d. The categorization of CD4 count into two groups only groups loses potentially valuable data. Will be better if considered e.g., quartiles or a continuous variable to give reveal additional trends.

**Response: A more granular categorization of CD4 counts could provide deeper insights into associations. However, such disaggregation may lead to information loss and reduced statistical power. Clinically, CD4 count is commonly categorized as <500 and ≥500 cells/µL to reflect immunological status, as lower counts (typically below 500 cells/µL) are associated with WHO clinical stage 3 opportunistic infections. We have therefore maintained the categorization for these reasons. However, we have performed a sensitivity analysis based on CD4 count categorization as <200 vs. ≥200 cells/µL and <350 vs. ≥350 cells/µL, and the results are shown in Table 4. Overall, we did not see statistically significant differences in the outcomes based on these categorizations.**

e. The study uses a cross-sectional QoL assessment, but the authors attempt to infer causality (suggesting that higher CD4 at initiation leads to lower mental QoL). A longitudinal design would be more appropriate for such conclusions.

**Response: The conclusion has been revised to show the findings are based on association but not causality in line with the study design. A longitudinal study was recommended for future research given the limitations of cross-sectional studies.**

f. Tables should be simplified with clear footnotes explaining abbreviations.

**Response: We have defined all abbreviations used in the table and written some of them in full to ease the readability.**

g. If possible, analyze QoL differences by gender, age group, or socioeconomic factors to add depth to findings.

**Response: We acknowledge this suggestion; however, the analysis of QoL by sex, age groups, and socioeconomic factors was not part of our a priori hypothesis or a pre-specified subgroup analysis. To ensure methodological transparency and minimize the risk of data-driven findings, we propose maintaining the study’s focus on its initial objectives and predefined hypotheses, however, these variables were adjusted for in the regression models.**

3. The manuscript states that all relevant data are fully available. A data availability statement is included, and the data seem to be within the manuscript and supporting files. However, The authors should consider depositing their dataset in a public repository with a DOI for transparency.

**Response: We acknowledge this suggestion. The journal guidelines allow data deposition through multiple avenues, including as supporting information. For this paper, uploading the dataset as supporting information aligns with the journal's requirements.**

4. The manuscript is generally well-written in standard English, but there are some grammatical errors, phrasing that can be improved and redundancies that need to be addressed.

a. Typographical errors noted in a number of areas. E.g lines 51-52: “Globally, there were 39 million people were living with human immunodeficiency virus (PLWH) by the end of 2022 [1].” Has a “were.” Highlighted that is unnecessary

**Response: The typing error has been removed. The revised text reads: “Globally, 39 million people were living with human immunodeficiency virus (PLWH) by the end of 2022”.**

**Additionally, the entire manuscript was proofread to identify and correct additional errors.**

Lines 53-54: “HIV is a treatable and preventable condition with life-long antiretroviral therapy (ART) and its scale-up has led to HIV becoming a manageable chronic health condition, enabling PLWH to live longer and healthier lives, almost comparable to people without HIV.”

**Response: This has been revised for clarity and it reads:**

**“HIV is a preventable and treatable condition but without a cure so requires life-long antiretroviral therapy (ART). The widespread scale-up of ART has transformed HIV into a manageable chronic health condition, allowing people living with HIV (PLWH) to achieve life expectancy and health outcomes similar to those in the general population.”**

The part of sentence “its scale-up has led to HIV becoming…” is off as it makes the sentence read like HIV scale up yet I believe reference is to ART thus could read better as “HIV is a treatable and preventable condition with lifelong antiretroviral therapy (ART). Its widespread use has transformed HIV into a manageable chronic condition, allowing PLWH to live longer and healthier lives, similar to those without HIV.” Line 282-283:

**Response: Thank you for this comment. We have revised the sentence as shown in the most immediate response (just above).**

“A study in South Africa revealed that factors such as age, sex, ART, and pill burden reduce the QoL [24].” To note is, “ART” is not a factor that reduces QoL; it should specify “ART-related side effects.”

**Response. We have corrected the error. We have used “ART-related side effects”.**

b. Some sentences are overly complex, making the manuscript harder to follow. For example, lines 69-72 “Early ART initiation (regardless of immune and clinical status) has several clinical and public health benefits such as preventing the progression of HIV to AIDS and preventing its transmission to other individuals as PLWH with undetectable viral load do not transmit HIV dubbed as Undetectable = Untransmittable or U=U [3, 7, 8].” Is too long with lots of information in one sentence, the phrase dubbed doesn’t sound good for a manuscript, and the “Undetectable = Untransmittable” (U=U) concept needs clearer introduction. Consider breaking it for example to read as “Early ART initiation, regardless of immune and clinical status, has multiple benefits. It helps prevent the progression of HIV to AIDS and reduces transmission. Research shows that PLWH with an undetectable viral load cannot transmit HIV, a concept known as ‘Undetectable = Untransmittable’ (U=U) [3,7,8].”

**Response: Thank you for the suggestion. We have adopted your suggested sentence. The revised sentence reads:**

**“Early ART initiation, regardless of immune and clinical status, has multiple benefits. It helps prevent the progression of HIV to AIDS and reduces transmission. Research shows that PLWH with an undetectable viral load cannot transmit HIV, a concept known as ‘Undetectable = Untransmittable.”**

c. Generally, edit as you simplify the long/complex sentences to improve readability.

**Response: We have reduced longer/complex sentences to improve readability.**

d. The results section repeats information that is already provided in tables, leading to redundancy. Consider having information in tables not repeated again in the narrative unless giving further details on the results. Instead of repeating full results, focus on key statistical takeaways.

**Response: Thank you for your feedback. We have revised the results section and retained only the essential statistics that enhance the readability of each section as a standalone component.**

e. Some references are outdated and should be replaced with recent studies (e.g., WHO 2024 guidelines).

**Response: We have updated the references using relevant citations.**
